# Implementation of point-of-care HbA1C instruments into community pharmacies: Initial development of a pathway for robust community testing

**DOI:** 10.1177/00045632231219380

**Published:** 2023-12-05

**Authors:** Andrew Radley, Lewis Beer, Danya Rushdi, Hazel Close, Stephen McBurney, Adrian Mackenzie, Anna Gourlay, Anna Barnett, Alison Grant, Neil Greig, Ellie Dow, Calum Sutherland

**Affiliations:** 1 School of Medicine, 85326 University of Dundee, Dundee, UK; 2 TASC, 85326 University of Dundee, Dundee, UK; 3 Blood Sciences, 1251 NHS Tayside, Dundee, UK; 4 Pharmacy Services, 3049 NHS Fife, Kirkcaldy, UK; 5 Pharmacy Services, 3129 NHS Lothian, Edinburgh, UK; 6 Pharmacy Services, 9561 NHS Borders, Edinburgh, UK; 7 NHS Research Scotland, Diabetes Network and School of Medicine, 85326 University of Dundee, Dundee, UK; 8 Diabetes Scotland, Glasgow, UK

**Keywords:** Type 2 Diabetes, Community Pharmacy, HbA1c testing, Point-of-Care

## Abstract

**Background:**

Point-of-care (POC) analysers in community settings can provide opportunistic and regular HbA1c monitoring. Community pharmacies in NHS Scotland are utilised by populations at greatest risk of type two diabetes (T2D). This study describes initial development of an HbA1c pathway using a POC analyser in community pharmacies.

**Methods:**

The Abbott Afinion analyser was compared in (i) NHS Tayside’s Blood Sciences Service and (ii) community pharmacies from four Scottish Health Boards. A side by side comparison with standard operating procedures for HbA1c quantification using 80 T2D patient venous samples. The machine was implemented into 11 community pharmacies and 144 samples obtained from patients for comparison to their recent laboratory HbA1c. Four focus groups examined themes around the intervention and an exit questionnaire was administered.

**Results:**

Laboratory assessment verified the efficacy of the POC test machine. The value for level 1 quality control was 44 mmol/mol and the mean during testing 42.7 mmol/mol. The greatest percent coefficient of variation (cv) was within-run for both levels of quality control material, at a value of 1.63% and 1.62%, respectively. The analyser performed robustly within the pharmacy assessment, with a mean difference of 1.68 and a standard deviation of 0.71 (CV 0.423). Patients with T2D reported positive experiences of using a pharmacy. The focus groups identified an appreciation of the convenience of pharmacies and of the longitudinal relationships with pharmacy staff.

**Conclusion:**

POC HbA1c analysers can be successfully established in community pharmacies. The target patient group responded positively to the opportunity to use a pharmacy service.

## Background

The prevalence of Type 2 Diabetes (T2D) is rising globally, with projections suggesting that the number of adults with the condition will increase from 415 million to 642 million between 2015 and 2040.^1^ Many cases, (∼175 million worldwide), are currently undiagnosed and perhaps 230 million people may have non-diabetic hyperglycaemia (NDH); a high-risk state for Type 2 Diabetes (T2D).^2^ For example, in Scotland, the number of people with T2D increased from 190,772 in 2008 to 267,615 in 2018^3^ with a greater burden falling on disadvantaged communities. The scale of this problem has led to population-based screening programmes such as the diabetes screening component of the National Cardiovascular Risk Assessment Programme in England, through general practices.^4^ Screening for high-risk groups is currently not performed in Scotland.

Early intervention can prevent development of diabetes and improve outcomes. There is large body of evidence showing that weight loss prevents conversion from prediabetes to diabetes.^5^ The challenge is to deliver early detection and intervention to people at risk of developing T2D, so that they have the opportunity to address this risk, without overburdening primary care.^6^ The Diabetes Remission Clinical Trial (DiRECT) demonstrated that a structured weight management programme can reduce risk, delay conversion to T2D and reverse the diagnosis and need for drugs.^7^ Other work has confirmed that the earlier the intervention, the greater the reduction in complications.^8^ Such interventions can be delivered in primary care and people can maintain the lifestyle changes for significant periods.^9^ Programmes across populations can prompt at-risk individuals to make beneficial lifestyle changes.^10^ The NHS now has validated weight loss options available but no cost-effective mechanism for identification of people who would benefit most from these available interventions.^11^

Many governments, including the Scottish Government, have put in place policies to increase the identification and treatment of people at high risk of T2D. Such policies promote sign posting of individuals to relevant information and referrals to services that might trigger early adoption of effective lifestyle change, (e.g. social prescribing, remote digital consultations and health coaching).^11^ Individuals who may benefit from such interventions can be identified through risk assessment tools.^12^ Such tools are now commonly used, both to guide assessment by health professionals and for public advice.^13^ The Diabetes UK risk tool has also been trialled within the Diabetes Prevention Programme in England.^14^ However, the available digital tools for diabetes risk were not developed as clinical aids but rather as a public health education and information platform. They are based on lifetime risk and have not yet been proven to accurately identify and stratify by immediate risk of progression to a requirement for clinical intervention. There are currently no population-based clinical screening programmes for those at highest risk of progressing to T2D, namely, those with prediabetic hyperglycaemia. The existence of validated diabetes prevention strategies within NHS Scotland make it even more important that these individuals are identified so that they can be offered the opportunity to engage with available programmes. As such, community pharmacies are present in each town and village and their significant reach into disadvantaged populations provides an unrivalled opportunity to target the highest risk population for progression to T2D.^15^

The diagnosis of T2D has primarily relied upon measurement of fasted plasma glucose levels and requires two separate measures above the threshold for risk of diabetes health complications, although HbA1c is now becoming the standard test for diagnosis.^16^ In community pharmacy settings, interpretation is complicated by the prandial status of the individual, as glucose homeostasis is affected by dietary intake.^17^ As an alternative, glycated haemoglobin (HbA1c) measurement is more suited to opportunistic assessment of glucose homeostasis, since levels reflect glucose management status over the last 90 days and does not require a second visit.^18^ Typically measurement of HbA1c has relied upon NHS laboratories to achieve the sufficient accuracy for clinical decision-making and came with the requirement for planned venepuncture. Measurement of HbA1c using CE-marked Point of Care (POC) testing equipment can resolve these issues and is of huge utility and benefit for convenient testing to obtain early diagnosis of prediabetes, risk of progression stratification, and regular monitoring of hard to reach patients (e.g. gestational diabetes), while reducing burden on GP clinic time, especially in a pandemic situation.^19^ The Abbott Afinion 2 instrument is a CE-marked device, with potential to provide robust and reliable HbA1c assays in POC situations.^20^

The work described within this manuscript verifies the performance of the Abbott Afinion 2 instrument against the regional laboratory standard. We then sought to establish whether community pharmacies could deploy this POC instrument to reliably ascertain HbA1c measurements from people diagnosed with diabetes. An initial process evaluation was commenced to gain an understanding of the views and perceptions of groups who would utilise the care pathway.

## Methods

### Study design

The study provides a head-to-head comparison of two analytical methods as part of the work to develop a community pharmacy point-of-care infrastructure, supported by effective governance systems. The first part of this evaluation compared the point-of-care instrument with the established laboratory method delivered by NHS Tayside’s standard procedure. The second part of the evaluation compared test results obtained from previously diagnosed patients with T2D, presenting at the pharmacy, to a recent HbA1c test obtained by the NHS laboratory as part of standard care.

A process evaluation was commenced to assess the acceptability of the intervention to participants. This first stage consisted of a focus group series drawn from a diverse participant group used to examine themes about the intervention. In addition, an exit questionnaire was employed for participants on leaving the pharmacy.

### Study protocol

Ethics approval was received for this study (21/YH/0281) from Yorkshire & The Humber – South Yorkshire Research Ethics Committee on 1 December 2021. Caldicott Guardian approval was given on 9 March 2022 and local Research and development permissions obtained for the participating Health Boards.

### In-laboratory validation of Abbott Afinion 2

#### Materials

The Abbott Afinion 2 is a fully automated POC device. It utilises Boronate Affinity Chromatography to measure glycated haemoglobin (HbA1c) in a 1.5 µL sample of venous or capillary whole blood. The reaction takes place within a HbA1c test cartridge, which contains the reagents required for the reaction. A sampling device, contained within the cartridge, is removed to collect the sample, before being returned to the cartridge. The test cartridge is placed in the Afinion, and once the door is closed, the reaction takes place. The sample is diluted and mixed with a reagent to release the haemoglobin from the red cells, allowing the haemoglobin to precipitate. The sample mixture is transferred to a well, containing boronic acid conjugate, which binds to the glycated haemoglobin. The reaction mixture is then soaked through a filter membrane, whereby, all precipitated haemoglobin, bound and unbound to the boronic acid conjugate, sticks to the membrane. Excess conjugate is removed with a wash reagent. The analyser measures the reflectance of the precipitate on the membrane, evaluating the intensities of the blue (glycated haemoglobin) and red (total haemoglobin) colours. The ratio between the two is proportional to the percentage of HbA1c in the sample, which is then displayed on the device screen.

#### Procedures

The verification of the analyser was performed in line with the NHS Tayside Blood Sciences Department Standard Operating Procedure, for the validation and verification of processes. As the Afinion 2 had already been validated by Abbott to CE standard, verification was required to ensure its performance was in line with the NHS laboratory service department’s acceptance criteria. Instrument performance was assessed and verified using within-run and between-run precision, analysis of External Quality Assurance (EQA) samples with assigned values and comparative studies of 80 patient venous samples randomly selected from routine care assessments. The range of these analysis is from 32 to 138 mmol/mol/. The measurement range of the instrument was 20–140 mmol/mol. Samples were stored at 4 C prior to analysis and were analysed in multiple batches within the 10-day stability window quoted by the manufacturer.

To assess within-run and between-run precision, replicate analyses of two quality control samples (one with a target value of 44 mmol/mol, and the other 66 mmol/mol) were performed, whereby, each sample was analysed five times per day, for the duration of 5 days in line with CLSI EP15-A3.

Six External Quality Assurance (EQA) samples, three samples from two different distributions of UK NEQAS for glycated haemoglobins, were analysed five times each, to allow assessment of accuracy. These results were compared against those of 35 other users of the same device, enrolled within the same EQA scheme.

The 80 patient samples were analysed on both the Afinion 2 and the laboratory gold standard method, the ADAMS ARKRAY HA-8180V (a reverse phase ion-exchange High Performance Liquid Chromatography [HPLC] method). Using this method, the fractions are separated by electrostatic interactions within the column gel, which contains hydrophobic and ion exchange groups on the surface. The separated haemoglobin fractions are detected at two wavelengths, 420 and 500 nm. The laboratory HbA1c method (Arkray) was enrolled in a ISO 17,043 accredited external quality assurance scheme and performance within the minimal analytical performance standard (MAPS), based on the European Biological Variability database hosted by the European Federation of Chemistry and Laboratory Medicine (EFLM) at https://biologicalvariation.eu/.^21^

Samples were randomly selected except for the age of sample being less than 3 days and exclusion criteria based on the Afinion 2 limitations, as stated by Abbott, which were:

• Patients with known haemoglobinopathies
• Those known to have HbF > 10%
• Those with haemolytic anaemia or other haemolytic diseases
• Pregnant patients

### In-pharmacy testing of Abbott Afinion 2

Afinion analysers were placed into 11 pharmacies, located across NHS Tayside (n = 3), Fife (n = 3), Lothian (n = 3) and Borders (n = 2). Potential participants were identified from the NHS Research Scotland (NRS) Diabetes Research Register, which is integrated into the national electronic health record, SCI Diabetes.

Appropriate training was given for all users of the Afinion 2, describing how to use it and the maintenance procedures. A training log was also created to outline the training required before use of the analyser, in addition to a formal record of user training, prior to use.

#### Inclusion

• Aged 18 or older
• People with Type 1 or Type 2 Diabetes
• Consent to participate in this study

#### Exclusion

• Participating in the intervention phase of another clinical study
• Absence or withdrawal of consent to participate

#### Procedures

The NRS Diabetes Research Register was used to identify potential participants and invite them to take part in the study. Potential participants were sent a Participant Information Leaflet (PIL) and details of where and when they could attend a pharmacy to take a test. Potential participants were only invited once and were free to decline by not responding to the invitation. A study contact number was provided to allow participants to ask any questions to a study coordinator before organising their appointment. Each patient was advised to attend the pharmacy within 2 weeks of their annual diabetic review in their general practice, from which a venous HbA1c had been measured.

When a participant attended a pharmacy, a member of the pharmacy staff trained on study procedures greeted the participant and obtained written consent. The pharmacy staff member obtained a capillary blood sample from the participant and analysed it using the Afinion instrument. The result of the HbA1c test was shared with the participant and recorded in the individual participant’s Case Report Form (CRF). Completed consent forms and CRFs were stored in the community pharmacy site file until the end of the study. A diabetes research nurse within each individual Health Board was asked to identify the laboratory-obtained HbA1c test of the participant, closest in date to the date of the pharmacy-obtained HbA1c test recorded on the CRF, and record this on the CRF.

The two groups of HbA1c results (pharmacy test and standard NHS laboratory test) were analysed using a paired two sample *t* test and assessment of precision (Levey–Jennings plot), test result agreement (Bland–Altman plot) and correlation (Passing–Bablock regression) as set out in the laboratory standard operating procedures.^22^ The precision of the pharmacy devices was compared by undertaking ANOVA analysis of pooled IQC performance (n = 42). Work was undertaken in Tayside by Clinisys to enable connectivity of the Afinion instruments through middleware (POCcellerator^©^) to the online laboratory test requests and results system, ICE. A system security policy was designed and full information governance permissions were obtained (DPIA). However, clinical results were not uploaded during recruitment to the study.

### Intervention acceptability

#### Procedures

A series of 4 focus groups were organised with support from Diabetes Scotland, a registered charity, using patient-partners already diagnosed with T2D. A purposive sample of participants was recruited from urban and remote/rural locations, as well as from groups with sensory disabilities and from ethnic groups with higher prevalence of type 2 diabetes. Group discussions explored the participants’ use of pharmacies, as well as their experiences of services and their opinions and preferences on having diabetes care provided from pharmacy teams. Interviews and focus groups were transcribed verbatim, noting pauses, laughter and outside noises using square brackets. Transcripts were analysed using reflexive thematic analysis.^23^ This method was chosen due to its flexibility which allows researchers to identify and compare themes in both sets of data and its suitability for large samples. The analysis was performed independently by AR and HC and reviewed by CS. Transcripts were coded using line-by-line coding. Codes were collated into themes by hand, using Microsoft Excel for data management. Field notes and analytical memos informed the coding. The groups’ discussions were used to inform construction of the exit questionnaire (Table 1).

**Table 1.** Patient feedback from using the pharmacy HbA1c service.

Question	Answers provided	Yes	No	Did not answer
1. How close was the pharmacy you visited to your home in miles?	≤1 mile = 5; between 1 and 5 miles = 74; between 6 and 10 miles = 18; greater than 10 miles = 41; unclear response = 5			
2. How did you travel to the pharmacy?	Car = 110; walk = 11; public transport = 20; unclear response = 2			
3. How close is the pharmacy you usually visit to your home in miles?	≤ Half a mile = 39; ≤1 mile = 47; 1–5 miles = 48; >5 miles = 8; unclear response = 1			
6. When you visited the pharmacy did the staff welcome you?		142	1	
7. Did the pharmacy staff understand the reason you were attending?		141	1	1
8. Were you shown into a private room in the pharmacy?		143		
9. Did the member of staff explain the test procedure?		143		
10. Did the member of staff seem confident in the test procedure?		143		
11. Did the member of staff tell you the result of the test?		142		1
12. Would you be happy to use the pharmacy again to get a test?		142	1	

1 participant did not answer any of the questionnaire.

All participants attending a pharmacy were invited to complete a structured exit questionnaire to ascertain acceptability of recruitment and their experience of using the pharmacy. Intervention participants were asked to rate the intervention, delivery, duration and likelihood of recommending the intervention to others.

## Results

### In-laboratory validation Abbott Afinion 2

#### Quantitative precision (ANOVA)

The values obtained from the within-run, between-run total (within-lab) precision testing are displayed in Table 2. The target value for level 1 quality control was 44 mmol/mol, and the mean value obtained during testing was 42.7 mmol/mol. The target value for level 2 quality control was 66 mmol/mol, with the testing mean at 63.6 mmol/mol. The total precision at both levels is consistent with those quoted by the manufacturer (1.6% for level 1 and 1.4% for level 2). This performance is within the 2.5% CV Minimum Analytical Performance Standards (MAPS) target as defined by the UK National Quality Assurance Advisory Panel (NQAAP). The values obtained for QC levels 1 and 2 were plotted in Levey–Jennings charts (Supplemental file - Figures 1 and 2, respectively) and showed that 88% of results fell within 1 standard deviation (SD) of the mean for level 1, and 72% fell within 1 SD and 24% within 2 SD for level 2. The findings of this section of the study are comparable with the manufacturer, Abbott’s, claim that the Afinion 2 analyser’s precision lies between 1 and 2 %CV.

**Table 2.** Table showing the values obtained from within-run and between-run precision testing.

					Within-Run		Between-Day		Within-Lab (Total)	
Analyte	Unit	Sample	Mean	N/outliers	SD	%CV	SD	%CV	SD	%CV
Abbott: HbA1c	mmol/mol	Level 1	42.7	25	0.663 (0.507–0.958)	1.55% (1.19%–2.24%)	0.21 (N/A)	0.491% (N/A)	0.696 (0.539–0.983)	1.63% (1.26%–2.3%)
		Level 2	63.6	25	0.775 (0.593–1.12)	1.22% (0.931%–1.76%)	0.684 (0.374–3.12)	1.07% (0.587%–4.9%)	1.03 (0.738–1.72)	1.62% (1.16%–2.71%)

#### Comparison

HbA1c values obtained for both the Afinion and the in-house assay (Arkray) were compared to evaluate the accuracy of the POC method (Table 2). The Afinion assay demonstrated a consistent negative bias in comparison to the laboratory standard assay, however, the Afinion results were consistent and stable across the range. Analyses of EQA material using the Afinion assay similarly identified a consistent negative bias, with significant bias and a significant difference between user and peer group (p < .001, Supplemental data).

The calculated values in Tables 3a and 3b were used to generate a Bland–Altman plot (Figure 3) and a Passing–Bablok regression chart (Figure 4). The Bland–Altman plot (Supplemental file - Figure 3) is used in the analysis of differences. Bland and Altman recommended that 95% of data points should lie within ±2SD of the mean difference.^21^ Figure 3 shows that 97.5% of values in this study fell within ±2SD of the mean difference between the two methods. Therefore, the instrument performs in line with accepted standards.

**Table 3a.** Table showing the statistical values calculated from the comparative the pharmacy results obtained using the Afinion 2 and the laboratory results obtained using the Arkray HA-8180V.

	Variable 1	Variable 2
Mean	57.09434	59.9434
Variance	156.4101	148.3777
Observations	106	106
Pearson correlation	0.967039	
Hypothesised mean difference	0	
df	105	
t stat	−9.20777	
*P* (T ≤ t) one-tail	1.83E-15	
t critical one-tail	1.659495	
*P* (T ≤ t) two-tail	3.67E-15	
t critical two-tail	1.982815	

t test: Paired two sample for means.

**Table 3b.** POC result (including + 3 mmol/mol correction factor).

	Variable 1	Variable 2
Mean	60.09434	59.9434
Variance	156.4101	148.3777
Observations	106	106
Pearson correlation	0.967039	
Hypothesised mean difference	0	
Df	105	
t stat	0.487829	
*P* (T ≤ t) one-tail	0.313344	
t critical one-tail	1.659495	
*P* (T ≤ t) two-tail	0.626688	
t critical two-tail	1.982815	

*t* test: Paired two sample for means.

From Table 2 and Figure 4 (Supplemental file), the regression line has a slope of 0.983 (0.957-1) and an intercept of −3.52 (−5 to −1.7). The correlation coefficient between both methods is r = 0.995, 95%CI 0.498 (−4.8448 to −3.852), P < .0001, with a strong relationship between the in-house method (Arkray HA8180-V) and Abbott Afinion 2. The Bland–Altman plot in Figure 5 (Supplemental file) shows a strong agreement between both methods, with approximately 96% of values falling within ±2SD of the mean.

### In-pharmacy testing of Abbott Afinion 2

The mean values obtained from pharmacy analysis agreed well with both target values and the laboratory comparison (Supplemental data). The total imprecision at both levels of QC was greater than that seen with the laboratory studies and the network performance is outwith the minimum criteria set by the IFCC.^22^ To our knowledge, we are not aware of a study that has reviewed the total imprecision across a network of multiple POCT HbA1c devices (n = 11), using non-laboratory staff, collecting multiple data points for over a four-month time period. As such, the performance we have observed may reflect more closely routine practice and has implications for how both quality control is monitored and performance ranges set for the intended clinical setting.^24^

144 HbA1c test results were obtained from the 11 pharmacies located within the Health Boards. 140 participants had a corresponding laboratory HbA1c result, with 106/144 having the lab test within the target 14 days of attending pharmacy. The mean time between tests was 11 days (range, 0–134 days) (Table 4). The three pharmacies in Tayside were commenced first and provided 78 tests; pharmacies in Fife provided 42 tests, pharmacies in Lothian provided 20 tests and pharmacies in Borders provided 4 tests, before the study was halted as having met its primary outcome. The SCI-Diabetes research database had much less coverage for NHS Borders than for the other participating boards.

**Table 4.** In-pharmacy testing participants.

	N = 140	Mean	SD	Range
Age		65.65	11.2	34–88
Gender (M/F)	M = 79 F = 61			
Type 1/Type 2	T1 = 33 T2 = 107			
Time from annual test (days)		11.46	12.90	0–134
Locality (miles)		8.3	N/A	N/A

A Bland–Altman plot and a Passing–Bablok regression chart were also constructed for the pharmacy arm of this work (Figure 5, Figure 6). The Bland–Altman plot in Figure 5 shows a strong agreement between both methods, with approximately 96% of values falling within ±2SD of the mean. The regression line (Supplemental file - Figure 6) has a slope of 1 (0.968–1.08) and an intercept of −3 (−7.39 to −1.58). The correlation coefficient between both methods is r = 0.97, 95%CI 0.61 (−3.61 to −2.39), *p* < .005, and there is therefore a strong relationship between the Arkray HA8180-V and Abbott Afinion 2 even though blood was drawn in different health care settings and the Arkray test sample was venous blood while the Abbott Afinion test sample was capillary blood.

Statistical analysis was performed on both sets of results (Table 3). A Kolmogorov–Smirnov normality test indicates that the difference in POC and NHS laboratory result follows normal distribution since, Dn = 0.108 < 0.131 = D_n, α_. The difference in test result between the POC testing (Mean = 57.094, SD = 12.506) and NHS laboratory testing (Mean = 59.943, SD = 12.181) was significant (t (106) = 1.98, p = 3.67 × 10^−15^), where the mean difference is −2.849.

Based on data from the laboratory verification work, it was hypothesised that the POC tests report a HbA1c measurement approximately 3 mmol/mol lower than the NHS laboratory test, with the batch of Afinion cartridges used. To test this, a 3 mmol/mol factor was added to each POC result. The difference in test result between POC testing (with the added 3 mmol/mol) (Mean = 60.094, SD = 12.506) and NHS laboratory testing (mean = 59.943, SD = 12.181) was not significant (t (106) = 1.98, p = .627). Reviewing the raw results obtained in the pharmacies identified that 4/144 patients would not have been identified as having an HbA1c of >42 mmol/mol if they had been assessed with the Afinion machine, compared to the laboratory standard (Positive Predictive Value 0.97). Similarly, from the raw results, 6/144 patients would have been identified as having an HbA1c of between 42 and 48 mmol/mol from the Afinion assay, but be identified as having a result of > 48.0 mmol/mol by the laboratory standard. This means that directly comparable results can be available in the clinical record, however, robust guidance for pharmacy colleagues needs to be in place to ensure that patients are not misclassified based on the immediate results available in pharmacies.

### Focus group outcomes

We conducted interviews with 14 participants across 4 focus groups. Discussions focussed on determining opinions related to use of Community Pharmacies in general and the services routinely accessed in the pharmacy setting, what was important to individuals when using a Community Pharmacy and their consideration of HbA1c testing being provided in this setting.

The vast majority of interviewees accessed their chosen Community Pharmacy for dispensing of their prescriptions, valuing the option of a collection and delivery service where offered. Accompanying medication advice was also a well-used service along with provision of treatment and advice for minor ailments.

Overwhelmingly what was valued the most in using Community Pharmacy for these services was the accessibility and locality close to home. The focus groups valued the pharmacy staff getting to know them and building a rapport, along with being helpful and knowledgeable, although it was recognised that when staff are busy and under pressure, this can impact negatively on these valuable interactions. A deaf-blind participant noted that special care was necessary when using the pharmacy accompanied by a support dog. A female participant of South Asian heritage noted some discomfort about using a pharmacy consultation room without a chaperone.

Examples of participants comments included:

*“... once you build that rapport everything else disappears as you have that trust in them.”*

*“getting advice sometimes was quite difficult because they were always quite harassed in the pharmacy”*

*Focus group members were keen to emphasise the need to have access to the Pharmacist and that the pharmacy staff have an understanding of their needs, such as social, cultural and disability awareness.*

*When asked about their thoughts on provision of diabetic testing through Community Pharmacy, each of the focus groups considered this to be a welcome service development. The focus groups suggested that this model may be a quick and easy option, and could lead to quicker results than usually received.*

*“I think it’s a good idea..... I would probably get more information and quicker which would give me a lot of peace of mind.”*

*“anything that .... helps the patient get quicker, faster more accurate results ..... is going to help.”*

*Focus group members welcomed the convenience of visiting a Community Pharmacy and that they hoped this would provide an opportunity to discuss the results and disease risks with the pharmacist.*

*“I would find the potential to go in and get that checked out at the pharmacy, I would find that quite convenient and reassuring.”*

*One focus group member raised concern regarding the additional workload that may be created for pharmacy staff, and advised that it would be important that the service would not create additional pressure that could jeopardise valued relationships and interactions.*

*“There has to be proper resourcing of the pharmacies for it ..... the pharmacists are busy .... I think we need to make sure the pharmacies are properly resourced.”*

*The focus groups were asked for their input in planning the diabetic testing pathway. Again, the importance of accessibility was valued the most, along with suggesting that an appointment system may help reduce pressure and ensure adequate time is spent with each individual.*

*“I think it is a positive step......as long as the pharmacy would do it by appointment .....rather than just turn up and be turned away.”*

*Ensuring that the testing was carried out in a private area was also a common theme mentioned in several focus groups.*

*“It is something that you would want to be done in private I think for most people.” Several focus group members agreed that clear communication regarding how the service worked was very important and that the service was provided by adequately trained staff that could provide expert advice.*

*“clear and concise communication so I know what is going on and they understand me and I understand them and then you get a good working relationship.”*

### Patient feedback from using the pharmacy HbA1c service

The exit questionnaire demonstrated a positive view of the pharmacy service provision, from this particular patient population. Almost all participants found the experience of attending a pharmacy acceptable (Table 4). All participants lived within a twenty-minute journey to their local pharmacy.

## Discussion

This programme of work has verified that a CE-marked POC analyser can achieve the performance required to operate within an NHS clinical environment and that accuracy can be successfully reproduced by pharmacy staff in a community environment. It is clearly possible to measure HbA1c with verified POC devices in the community with an analytical quality similar to current gold standard clinical laboratory procedures.^21^ Our work demonstrates that directly comparable results can be produced in a pharmacy environment and can be incorporated into the clinical record, though precision studies across a network of devices highlight the ongoing need for laboratory oversight of QC performance and robust management of both reagent and QC lot variation. This assessment has enabled us to have confidence in the precision and reliability of the point-of-care approach.

Until recently, population wide HbA1c screening was deemed unfeasible and unnecessary. This was because standard approaches were expensive, time consuming for staff, would require dedicated regular primary care visits for large numbers of individuals with no clinical symptoms and there were no validated diabetes prevention programmes available to offer those with elevated non-diabetic HbA1c levels. This situation changed with evidence that; (1) HbA1c levels between 42 and 48 mmol/mol are a prediabetic stage that comes with a significantly higher risk of progressing to symptomatic T2D, (2) delaying the onset of T2D delays the onset of diabetes complications and the associated morbidity and mortality and (3) successful weight management delays the progression of HbA1c elevation and thus provides successful diabetes prevention. For these reasons, the main piece of major diabetes prevention programmes still missing is the cost-effective identification of the asymptomatic high-risk prediabetic population who would benefit most from available validated weight management programmes. Providing interventions for all those overweight or obese is simply unaffordable as 67% of the adult Scottish population falls into this category. Importantly, the vast majority population may be unaware of the significance of their status but are not in imminent danger of progression to T2D, although raising awareness may still be a worthwhile goal for them. Accurate monitoring of HbA1c in the asymptomatic population would provide key clinical information to identify those (with rising HbA1c) in greatest need of robust diabetes prevention intervention. We propose that the creation of a community pharmacy infrastructure has the potential to increase the capacity of primary care services and make services available to groups who may struggle to access established services. ^13, 15^ Our initial work indicates that such a service would be acceptable to patients.

The use of POC HBA1c testing has a series of additional clinical advantages. When there is access to POC HbA1c testing, more people are likely to receive a HbA1c test and the number of patients with documented HbA1c increases.^25^ POC HbA1c testing can also facilitate the identification of undiagnosed ‘missed’ T2D, a current unmet clinical need.^26–28^ Robust POC HbA1c tests in the community would have applications beyond T2D prevention. As an option for routine diabetes services, it would improve disease management. It has the potential to engage harder to reach communities, as well as provide a convenient option for women with a history of gestational diabetes (the highest risk group for T2D) to allow regular monitoring of their glucose homeostasis. Some studies have used this technique to support lifestyle intervention and engagement in care.^29,30^ T1D and T2D patients with access to POC testing may opt to get tested more frequently and therefore may gain improved glycaemic control.^28–30^ Similarly, the time between testing and follow-up can decrease.^31,32^ Patients are positive about the option of POC testing and value the immediate availability of results and the removal of a requirement to return at a later date.^33^ A value-based case establishing POC testing within community pharmacy practice would enable care pathways for several different aspects of diabetes care (gestational diabetes, diabetes annual checks) and cardiovascular care to be delivered and standards of care met, as a team contribution within primary care, as part of NHS strategy to deliver more integrated care.^34^

A key strength of this work programme has been the close partnership with NHS Blood Sciences colleagues, to verify the performance of a POC instrument and advise on the construction of a quality framework. There has been caution in adopting widespread POC testing, due to the variable quality of different devices and the resources required to successfully operate the devices within a quality environment.^35,36^ In the UK, the Medicine and Healthcare Products Regulatory Agency (MHRA) Guidance states that ‘The local hospital pathology laboratory should play a key role in the development and management of a POC service’. This is particularly true for secondary care and may also be useful for some primary care services. ^35^ While many UK laboratories provide support for GP and community POC testing, there is little information available on the extent of involvement. There is certainly no formal national POC testing support structure in place and what support does exist may be ad-hoc and variable in extent and quality. It is acknowledged that this current programme of work describes an early stage of implementation and many challenges have yet to be addressed, as we scale up and spread the extent of the community pharmacy infrastructure, within a systematic quality framework. An extensive health economic case is still required but this will be facilitated by the development of additional CE-marked POC instruments. To provide seamless and safe data connectivity with local digital health records is a priority. This can be achieved with this infrastructure.

A number of further developments are now desirable, including confirmation that people identified in pharmacies can be successfully linked to other key services, such as weight management services for prediabetics, or referral to general practices, if overt diabetes is indicated. In Scotland, electronic professional-to-professional communication is established, which facilitates clinical communication. The potential utility of community pharmacies developing pathways for chronic disease management, as part of the independent prescribing capability, and providing much needed support to GP practise, is an emerging area that will generate much interest.^37^

## Conclusion

It is possible to establish a POC analyser in real world community health care settings that can achieve the precision required to operate to NHS clinical laboratory standards. An analyser such as the one used in this study can be successfully operated by pharmacy staff in a community environment and provide equivalent robust HbA1c measures to current gold standard laboratory pathways. In addition, a potential target patient group responded positively to the opportunity to use a pharmacy service to access HbA1c testing with immediate test result feedback.

## Supplemental Material

Supplemental Material - Implementation of point-of-care HbA1C instruments into community pharmacies: Initial development of a pathway for robust community testing Supplemental Material for Implementation of point-of-care HbA1C instruments into community pharmacies: Initial development of a pathway for robust community testing by Andrew Radley, Lewis Beer, Danya Rushdi, Hazel Close, Stephen McBurney, Adrian Mackenzie, Anna Gourlay, Anna Barnett, Alison Grant, Neil Greig, Ellie Dow, and Calum Sutherland in Annals of Clinical Biochemistry.

## Data Availability

All data used in this study is contained within the manuscript.
