# Supplementary material for: Implementation of point-of-care HbA1C instruments into community pharmacies: Initial development of a pathway for robust community testing
Source: Ann Clin Biochem. 2023 Dec 5;61(4):273–83. doi: 10.1177/00045632231219380 (PMC11337724; doi:10.1177/00045632231219380)
Supplement: Supplemental Material - Implementation of point-of-care HbA1C instruments into community pharmacies: Initial development of a pathway for robust community testing [file sj-pdf-1-acb-10.1177_00045632231219380.pdf]

Supplementary File

Figure 1. Levey-Jennings Chart plotting the precision test values for QC Level 1 (series 1-5 represents days 1-5)

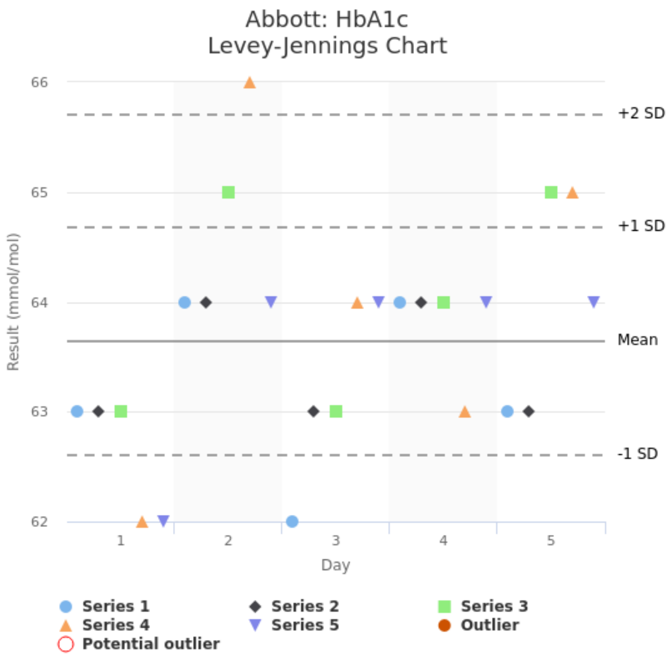

Figure 2. Levey-Jennings chart plotting the precision test values for QC Level 2 (series 1-5 represents days 1-5)

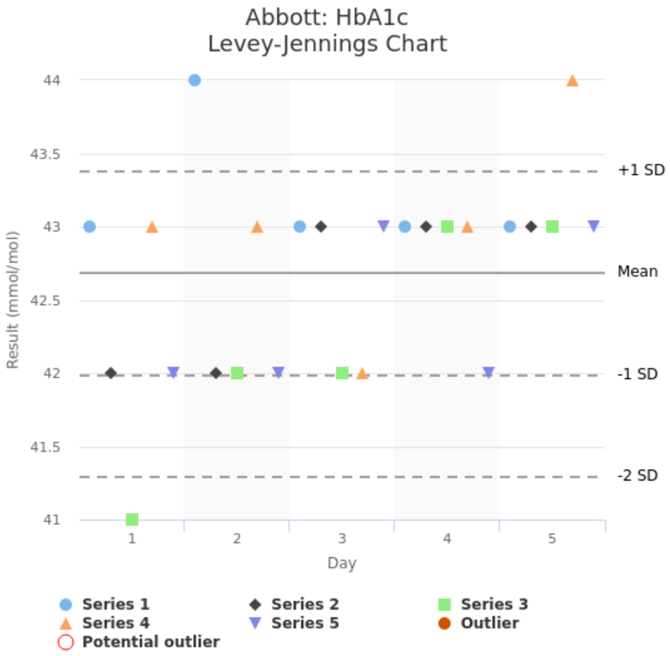

Figure 3. Bland Altman Plot describing the agreement between the Arkray HA8180-V and the Abbott Afinion 2.

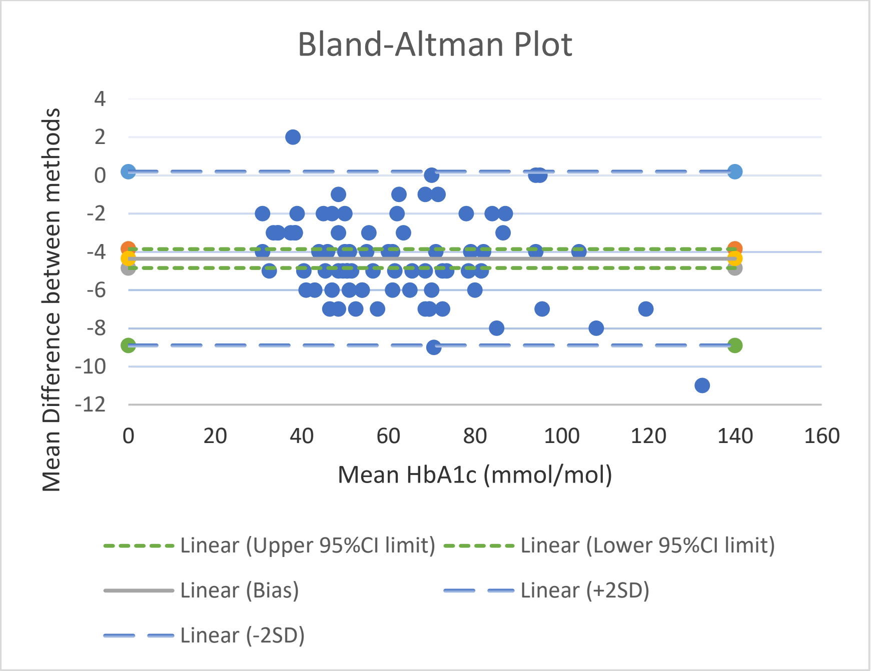

Figure 4. Passing-Bablok linear regression showing the correlation between Arkray HA8180-V and Abbott Afinion 2.

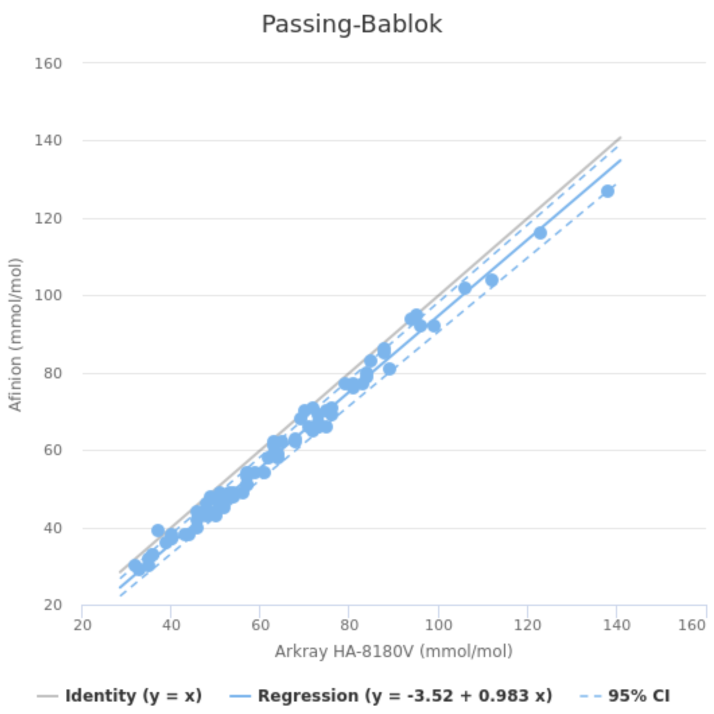

Figure 5 Bland Altman Plot showing the agreement between HbA1c measurement on the Afinion 2 within the pharmacy setting and the laboratory, using the Arkray HA-8180V.

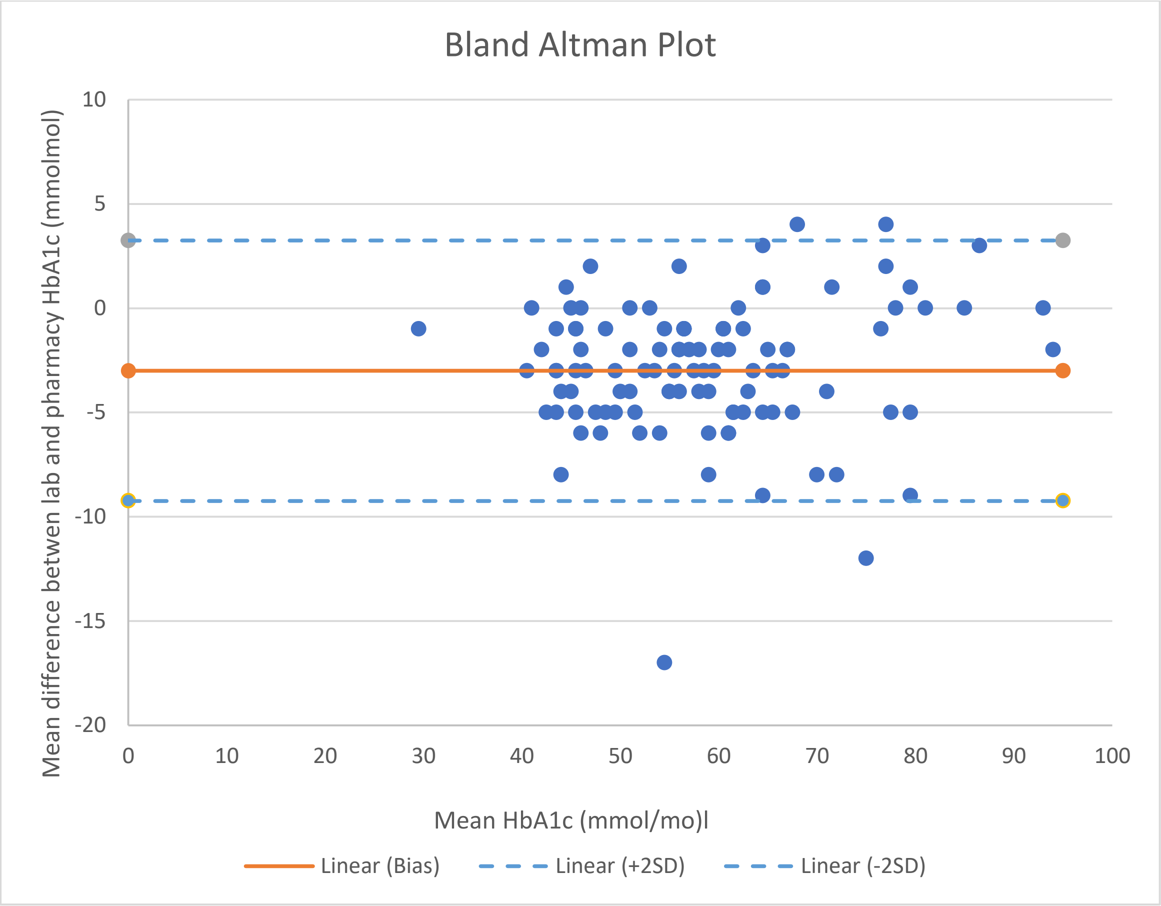

Figure 6 Passing-Bablok linear regression showing the correlation between the Afinion 2 within the pharmacy setting and the laboratory Arkray HA-8180V

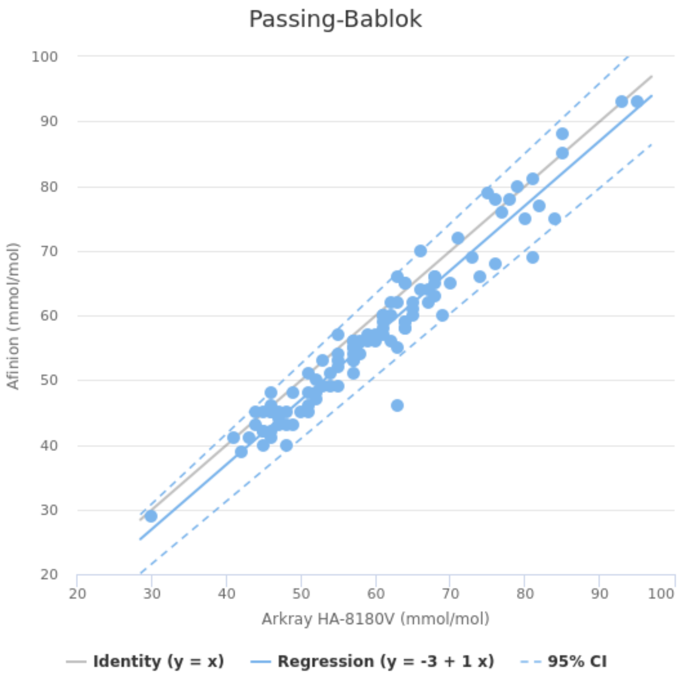

Table 1 – Anova summary of Afinion EQA comparison

Average Z-score	-1.39	Peer group average:	46.17
S(Z)	0.41	SEM_{Peer}	4.44
SEM_z	0.17	Average user results:	43.72
t_{crit}	2.571	SEM_{user}	4.08
t_{dep}	-8.341	Diff:	2.5
df	5	t_{crit}	2.57
α	5 %	t_{dep}	-6.765
p-value (two sided)	0.000	α	5 %
Bias different from zero		p-value (two sided)	0.001
		User and peer group different	

Table 2 – Summary of Pharmacy precision studies

					Within-Run		Between-Day		Within-Lab (total)	
Analyte	Unit	Sample	Mean	N / Outliers	SD	%CV	SD	%CV	SD	%CV
Afinion HbA1c	mmol/ mol	Level 1	42.9	42	1.69 (1.38 – 2.18)	3.94% (3.22% – 5.08%)	1.81 (0.995 – 8.1)	4.23% (2.32% – 18.9%)	2.48 (1.69 – 4.61)	5.78% (3.95% – 10.8%)
		Level 2	64.3	42	3.28 (2.68 – 4.23)	5.1% (4.17% – 6.58%)	0.606 (N/A)	0.942% (N/A)	3.34 (2.74 – 4.28)	5.19% (4.25% – 6.65%)
